# Primary thyroid B‐cell lymphoma: molecular insights into its clonal evolution and relapse

**DOI:** 10.1002/path.6380

**Published:** 2024-12-26

**Authors:** Maria‐Myrsini Tzioni, Natsuko Watanabe, Zi Chen, Fangtian Wu, Ewelina Madej, Jasmine Makker, Sarah Guo, Ayoma D Attygalle, Andrew Wotherspoon, Kiminori Sugino, Koichi Ito, Ming‐Qing Du

**Affiliations:** ^1^ Department of Pathology University of Cambridge Cambridge UK; ^2^ Department of Internal Medicine Ito Hospital Tokyo Japan; ^3^ Department of Histopathology Royal Marsden Hospital London UK

**Keywords:** thyroid lymphoma, lymphoma relapse, Hashimoto's thyroiditis, mutation profile, clonal evolution

## Abstract

Primary thyroid lymphomas comprise largely extranodal marginal zone lymphoma of mucosa‐associated lymphoid tissue (EMZL) and diffuse large B‐cell lymphoma (DLBCL), followed by follicular lymphoma (FL). They commonly develop from a background of Hashimoto's thyroiditis (HT), where dysregulated immune responses trigger autoreactive infiltrates and drive clonal B‐cell evolution. To understand how these lymphomas and their relapse evolve, we investigated 10 cases by mutation profiling, including five with metachronous lymphomas [primary lymphoma (EMZL = 4, DLBCL = 1) with local relapse (EMZL = 3, DLBCL = 2)], one composite EMZL and Epstein–Barr virus (EBV)‐positive DLBCL, and four lymphomas (EMZL = 3, FL = 1) with prior or subsequent biopsy showing HT. In four cases with metachronous lymphomas, both common and distinct variants were seen in the paired lesions, indicating their divergent evolution from clonally related lymphoma precursor (CLP) cells. In the remaining case with metachronous lymphomas, the relapsed lesion was progressed from the initial lymphoma. In the case with composite lymphoma, the EBV‐positive DLBCL was transformed from EMZL. Finally, in the four cases with paired lymphoma and HT biopsies, two showed shared mutations between the paired lesions, indicating involvement and divergent evolution from CLP cells. Thyroid lymphoma relapse may frequently develop via divergent evolution from a CLP cell, which is likely premalignant. © 2024 The Author(s). *The Journal of Pathology* published by John Wiley & Sons Ltd on behalf of The Pathological Society of Great Britain and Ireland.

## Introduction

Lymphoma development is a dynamic evolutionary process involving a stepwise acquisition of multiple cooperating events including both somatic genetic changes and dysregulated immune responses. These events drive the selection of certain reactive B cells, their clonal expansion, and eventual malignant transformation. Theoretically, the evolutionary process may generate a pedigree of clonal B cells with variable malignant potentials depending on the extent of cooperative oncogenic events in individual subclones. This is best exemplified in the lymphomagenesis driven by t(14;18)(q32;q21)/*IGH*::*BCL2*, which occurs in pre‐B cells due to erroneous *IGH* VDJ recombination and causes constitutive expression of BCL2. Overexpression of BCL2 prevents the translocation‐positive B cells from commencing apoptosis during the germinal centre reaction in the peripheral lymphoid tissues, thereby causing their clonal expansion and the formation of *in situ* follicular B‐cell neoplasia (ISFN). As the *IGH*::*BCL2*‐positive B cells transit relentlessly from one B‐cell follicle to another, they are at risk of acquiring genetic changes, particularly due to the off‐target effects of the somatic hypermutation machinery [1, 2]. With sufficient cooperative oncogenic events, this will lead to malignant transformation, developing an overt follicular lymphoma (FL). As the premalignant *IGH*::*BCL2*‐positive B‐cell clone persists and continues to expand through the germinal centre reaction, this may result in additional malignant transformation, giving rise to a new lymphoma, such as transformed FL or relapsed FL, and occasionally also other low‐grade B‐cell lymphomas [3, 4, 5, 6, 7, 8]. This has been known as divergent evolution, explaining at least in part why FL is incurable despite being an indolent low‐grade tumour [9]. Such dynamic lymphoma evolutionary process initiated by *IGH*::*BCL2* provides a paradigm for investigations of clonal evolution in other lymphomagenic conditions.

Another well‐recognised multistage lymphomagenic process is the development of extranodal marginal zone lymphoma of mucosa‐associated lymphoid tissue (EMZL), which invariably arises from a background of a chronic inflammatory disorder due to bacterial infection (such as *Helicobacter pylori*‐associated gastritis) and autoimmunity [Hashimoto's thyroiditis (HT), Sjögren's syndrome] [10]. These prolonged inflammatory processes provide a setting for evolution of ‘lymphomagenic’ (often autoreactive) B cells, with their clonal expansion and eventual malignant transformation following acquisition of sufficient cooperative somatic genetic changes. Although the molecular mechanisms driving the oncogenic process in EMZL development are very different from those in FL, the premalignant clonal B‐cell population generated in such chronic inflammatory disorders may also confer multimalignant potential. To address this, we investigated a series of primary thyroid lymphomas with consecutive biopsies that showed local lymphoma relapse or prior/subsequent HT.

## Materials and methods

### Patients and clinical data

The use of archival tissues for research was approved by the ethics committees of the institutions involved.

**Figure 1:**
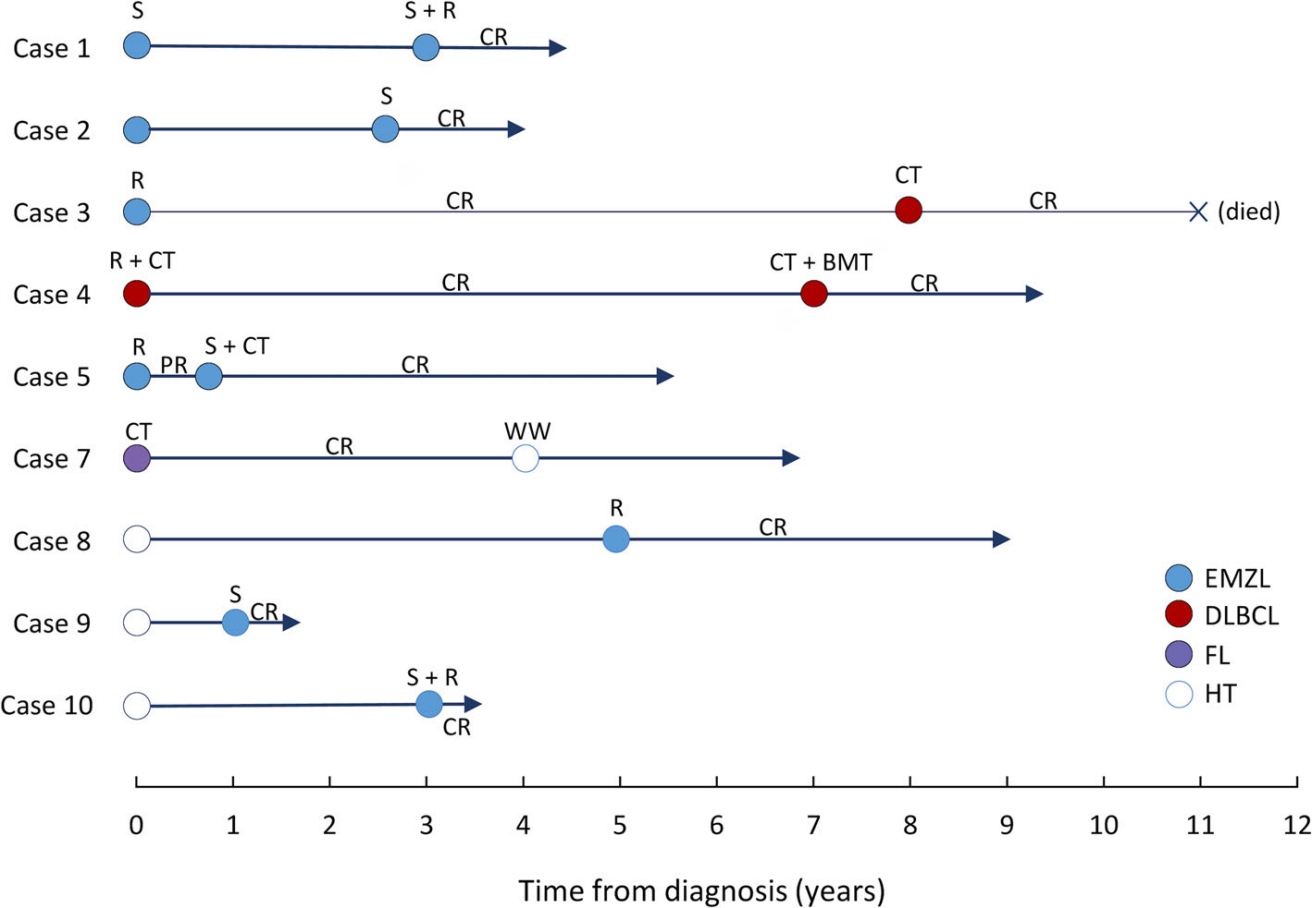
Summary of primary thyroid lymphoma and clinical follow‐up data. CR, complete remission; CT, chemotherapy; DLBCL, diffuse large B‐cell lymphoma; EMZL, extranodal marginal zone lymphoma of mucosa‐associated lymphoid tissue; FL, follicular lymphoma; HT, Hashimoto's thyroiditis; PR, partial remission; R, radiation therapy; S, surgical resection; WW, watch and wait.

**Figure 2:**
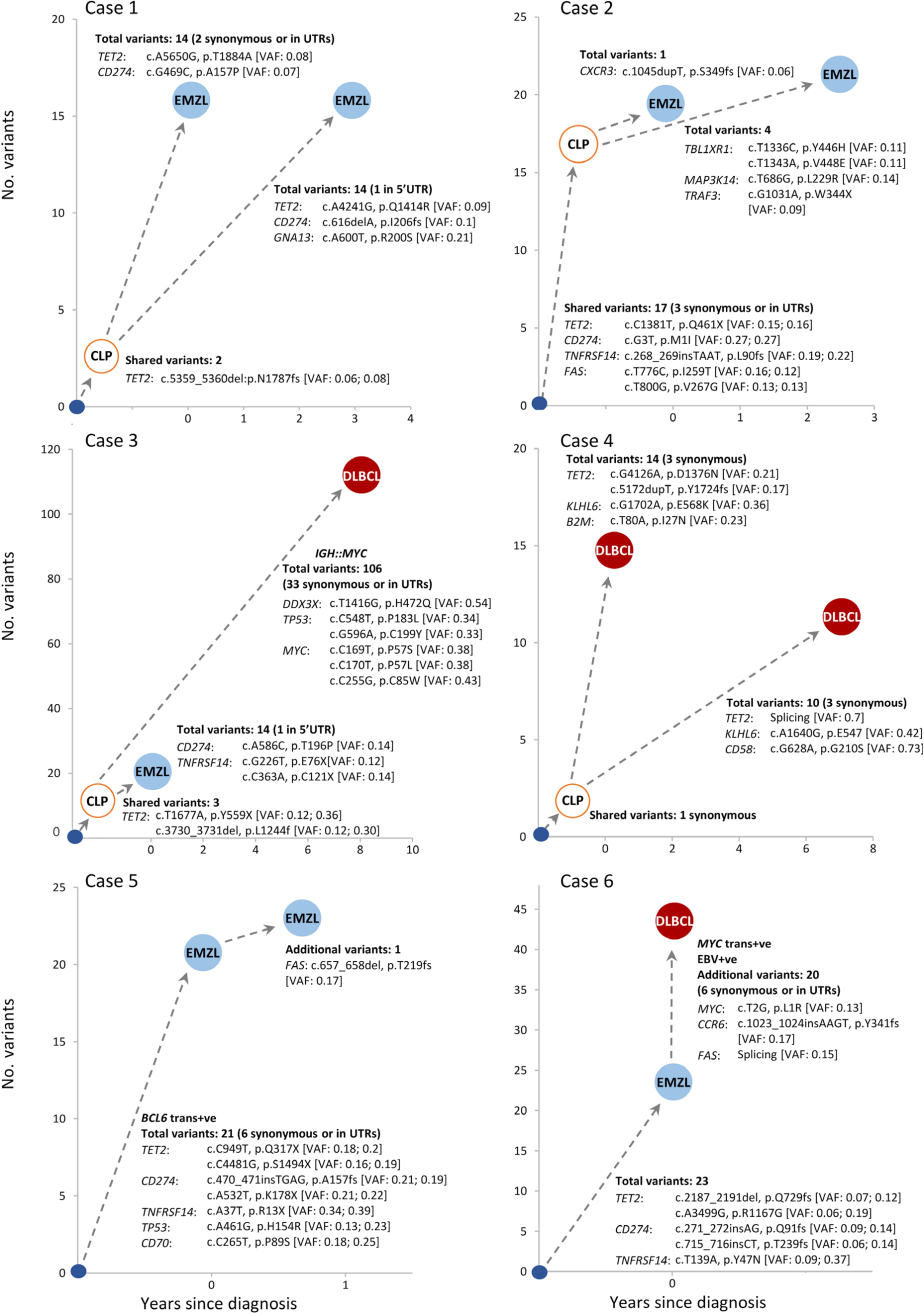
Variants in metachronous thyroid lymphomas or composite lymphomas. The number of shared and distinct clonal variants in paired lesions and their predicted evolutionary trajectory in each case are indicated. The total number of all clonal variants (including pathogenic, benign, synonymous variants, and those in UTR regions) is given, but only representative pathogenic mutations are shown in detail. CLP, predicted clonally related lymphoma precursor cells; DLBCL, diffuse large B‐cell lymphoma; EMZL, extranodal marginal zone lymphoma of mucosa‐associated lymphoid tissue; FL, follicular lymphoma; trans+ve: translocation positive; VAF, variant allele frequency, for variants shared by paired lesions and so thought to occur in the CLP cells; the two VAF values in brackets correspond to the paired lesions in sequence.

Ten cases of primary thyroid lymphoma with consecutive thyroid tissue biopsies were studied (Figures 1 and 2, Table 1), and their histological diagnosis was reviewed. The final diagnosis comprised five cases of metachronous lymphomas [primary lymphoma (EMZL = 4, DLBCL = 1), with relapse or high‐grade transformation 10 months to 8 years later (EMZL = 3, DLBCL = 2)], one case of composite EMZL and Epstein–Barr virus (EBV)‐positive DLBCL, and four cases of lymphoma (EMZL = 3, FL = 1) with prior or subsequent biopsy showing HT. Formalin‐fixed paraffin‐embedded (FFPE) tissue specimens were available for each case.

**Table 1:** Summary of clinical and laboratory results of cases investigated.

Thyroid lobe involved	Diagnosis	Clinical stage	Serology	Immunophenoytpe	NGS‐based *IG* clonality analysis	Genetic changes and tumour clonal evolution*		Treatment	Clinical outcome
**Case 1**: 67‐year‐old female with 5‐year history of HT									
Right lobe	Original diagnosis of HT, but EMZL on review	IE	TgAb+ TPOAb+	CD10−, BCL6−, BCL2+	Clonal *IGHV4‐34/JH6* (FR1‐JH primers)	*BCL2* trans−ve *BCL6* trans−ve	Shared mutations: 2 Unique mutations: 14 in first lesion, 14 in second lesion	Right lobectomy	Progressed to EMZL 36 months later
Left lobe	EMZL	IE	TgAb+	CD10−, BCL6−, BCL2+	Failed to amplify	*BCL2* trans−ve *BCL6* trans−ve	Evolution: divergent from clonally related lymphoma precursor cells	Total residual resection and local radiation (30.6 Gy)	CR and alive at last follow‐up (15 months after treatment)
**Case 2:** 59‐year‐old female with 2‐year history of HT									
Left lobe	Original diagnosis of HT, but EMZL on review	IE	TgAb+ TPOAb+	CD10−, BCL6−	Identical clonal *IGKVD3‐20*/*JK2* rearrangement (*IGK* Tube A)	*BCL2* trans−ve *BCL6* trans−ve	Shared mutations: 17 Unique mutations: 1 in first lesion, 4 in second lesion	n/a	Open biopsied (resection), otherwise no other active treatment, but progressed to EMZL 31 months later
Both lobes	EMZL	IE	TgAb+ TPOAb+	n/a		*BCL2 trans − ve* *BCL6 trans − ve*	Evolution: divergent from clonally related lymphoma precursor cells	Total thyroidectomy	CR and alive at last follow‐up (18 months after treatment)
**Case 3:** 76‐year‐old female with 6‐year history of HT									
Right lobe	EMZL	IE	TgAb+ TPOAb+	CD10−, BCL6−, MYC+ (<5%)	Failed to amplify	*BCL2* trans−ve *BCL6* trans−ve *MYC* trans −ve	Shared mutations: 3 Unique mutations: 14 in first lesion, 106 in second lesion	Local radiation (38 Gy)	Achieved CR, but relapse 8 years later
Right lobe	DLBCL	IIE	TgAb‐ TPOAb+	CD10−, BCL6+, MUM1−, MYC+ (~70%)	Clonal *IGHV3‐15/JH4* rearrangement (FR2‐JH primers)	*BCL2* trans−ve *BCL6* trans−ve *IGH*::*MYC* trans +ve	Evolution: divergent from clonally related lymphoma precursor cells	R‐CHOP × 6	Achieved CR, died of infection due to MDS 36 months later
**Case 4:** 57‐year‐old male with HT diagnosed together with initial lymphoma									
Right lobe	DLBCL	IIE	TgAb+	CD10+, BCL6+, BCL2+, MUM1−, MYC+ (~50%)	Identical clonal *IGHV3‐7*/*JH4* rearrangement (FR2‐JH primers)	*BCL2* trans−ve *BCL6* trans−ve	Shared mutations: 1 Unique mutations: 14 in first lesion, 10 in second lesion	Local radiation (36 Gy) and R‐CHOP	Achieved CR, but lymphoma relapsed 7 years later
Left lobe	DLBCL	IIIE	TgAb+ TPOAb‐	CD10+, BCL6+, MYC+ (~15%), MUM1−		*BCL2* trans−ve *BCL6* trans−ve	Evolutionary: divergent from clonally related lymphoma precursor cells	R‐ICE × 2, followed by BMT	Achieve CR, alive at last follow‐up (29 months after treatment)
**Case 5**: 65‐year‐old female with no previous history of HT									
Left lobe	EMZL	IE	TgAb− TPOAb−	CD10−, BCL2+, BCL6−	Failed to amplify	*BCL2* trans−ve *BCL6* trans+ve	Shared mutations: 21 Unique mutations: 1 in second lesion	Local radiation (36 Gy)	Achieved PR, but disease progressed 10 months later
Right lobe	EMZL	IE	TgAb+ TPOAb‐	CD10−, BCL6−, BCL2+		*BCL2* trans−ve *BCL6* trans+ve	Evolution: linear progression	Total thyroidectomy & R‐CHOP x 3	Achieved CR, alive at last follow‐up (57 months after treatment)
**Case 6**: 60‐year‐old female with 39‐year history of HT, positive for EBV									
Right lobe	EMZL	IIE	TgAb+ TPOAb+	CD10−, BCL6+, MYC+ <5%, EBER negative	n/a	*BCL2* trans−ve *BCL6* trans−ve *MYC* trans−ve	Shared mutations: 23 Unique mutation: 20 in second lesion	R‐CHOP (number of cycle unknown)	CR and alive at last follow‐up (39 months after treatment)
	DLBCL			CD10−, BCL6+, MYC+ (~30%), EBER positive		*BCL2* trans−ve *BCL6* trans−ve *MYC* trans+ve	Evolutionary: linear progression		
**Case 7**: 73‐year‐old female with 4‐month history of HT									
Both lobes	FL3A	IIE	TgAb+ TPOAb‐	CD10−, BCL6+, BCL2+	n/a	*BCL2* trans−ve *BCL6* trans−ve	Shared mutations: 1 Unique mutation: 28 in first lesion, 1 in second lesion	R‐CHOP×3	Achieved CR, then suspicious lymphoma relapse 48 months later
Both lobes	HT	n/a	TgAb+	n/a		*BCL2* trans−ve *BCL6* trans−ve	Evolutionary: divergent from clonally related lymphoma precursor cells	Watch and wait	Alive at last follow‐up (35 months after treatment)
**Case 8:** 62‐year‐old female with 2‐year history of HT									
Left lobe	HT	n/a	TgAb+ TPOAb+	n/a	Identical clonal *IGKV1‐12*/*KDE* rearrangement (*IGK* tube B)	n/a	Shared mutation: 8 Unique mutation: 9 in first lesion, 10 in second lesion	n/a	Progressed to EMZL 5 years later
Right lobe	EMZL	IE	n/a	CD10−, BCL6−		*BCL2* trans−ve *BCL6* trans−ve	Evolutionary: divergent from clonally related lymphoma precursor cells	Local radiation (34 Gy)	CR and alive at last follow‐up (48 months after treatment)
**Case 9**: 71‐year‐old female with a 30‐year history of HT									
Left lobe	HT	n/a	TgAb‐ TPOAb+	n/a	n/a	n/a	No mutations identified	n/a	Progressed to EMZL 12 months later
Left lobe and small low echoic spots in right lobe	EMZL	IE	n/a	CD10−, BCL6−		*BCL2* trans−ve *BCL6* trans−ve	28 unique clonal mutations	Total thyroidectomy	CR and alive at the last follow up (8 months after treatment)
**Case 10**: 73‐year‐old female with no previous history of HT									
Left lobe	HT	n/a	TgAb+ TPOAb+	n/a	n/a	n/a	No mutations identified	n/a	Progressed to EMZL 36 months later
Left lobe	EMZL	IIE	TgAb+ TPOAb+	CD10−, BCL6−, BCL2+		*BCL2* trans−ve *BCL6* trans+ve	59 unique clonal mutations	Total thyroidectomy and local radiation (50Gy)	CR and alive at last follow‐up (7 months after treatment)

Abbreviations: BMT, bone marrow transplantation; CR, complete remission; DLBCL, diffuse large B‐cell lymphoma; EBV, Epstein–Barr virus; trans+ve: translocation positive; trans−ve: translocation negative; EMZL, extranodal marginal zone lymphoma of mucosa‐associated lymphoid tissue; FL, follicular lymphoma; HT, Hashimoto's thyroiditis; MDS, myelodysplastic syndromes; n/a, not available; PR, partial response; TgAb, thyroglobulin antibody; TPOAb, thyroid peroxidase antibody.

* Only clonal variants with a variant allele frequency (VAF) >0.05 were included for lymphoma lesions, while all variants, including those seen in paired lymphoma specimens regardless of their VAF, were considered for the HT lesion.

### Immunohistochemistry and interphase fluorescence *in situ* hybridisation (FISH)

Immunohistochemistry and EBV‐encoded RNA *in situ* hybridisation were performed on FFPE tissue sections using an automated Bond‐III system (Leica Biosystems, Newcastle upon Tyne, UK) with Bond Polymer Kit (supplementary material, Table S1). *BCL2*, *BCL6*, and *MYC* translocations were investigated on FFPE tissue sections by interphase FISH using Vysis probes (Abbott Molecular, Des Plaines, IL, USA).

### DNA extraction and quality assessment

For each lymphoma specimen, tumour‐cell‐rich areas (>30%) were microdissected from FFPE tissue slides. DNA was extracted using the QIAamp DNA Micro Kit (QIAGEN, Manchester, UK), quantified using a Qubit® Fluorometer (Life Technologies, Paisley, UK), and assessed for quality using PCR generation of variable sized genomic fragments [6].

### Targeted next‐generation sequencing (NGS)

A panel of 275 genes was investigated for mutation by targeted NGS (supplementary material, Table S2). A total of 100–200 ng DNA were fragmented using a Covaris E220 Focused Ultrasonicator (Covaris, Brighton, UK). For each DNA sample, an indexed library was prepared with the xGen™ UDI‐UMI indexes (IDT, Coralville, IA, USA) and then pooled for target enrichment using TWIST probes (TWIST Biosciences, South San Francisco, CA, USA) [11]. The enriched gene targets were amplified using PCR, and pooled libraries were sequenced using the NextSeq 2000 platform (Illumina, San Diego, CA, USA) and the 2×100 bp paired‐end sequencing protocol. The sequence data analysis, variant calling, and filtering were performed as described in previous studies [6, 11]. Variants in lymphoma lesions with a VAF of >0.05 were considered clonal and filtered for SNPs with a minor allele frequency of ≥0.01%. Variants identified in any lesion and their potential presence in the paired biopsy were verified by checking bam files using the Integrative Genomics Viewer (version 2.12.3) [12].

### Clonality analysis of rearranged immunoglobulin heavy chain genes (IGH)

This was performed using the BIOMED‐2 assays, followed by NGS (Illumina NovaSeq X Plus sequencer using a 150‐bp end sequencing protocol) [1].

## Results

Targeted NGS for mutation profiling was successful for each specimen, as shown by the adequate sequencing coverage (>99% with a minimum of 100 reads after deduplication) and sequence read quality (supplementary material, Figures S1 and S2). All somatic variants, including pathogenic, benign, and synonymous changes, and variants in untranslated regions (5’‐UTR, 3’‐UTR) were compared between the paired lesions to establish their clonal relationship and evolutionary trajectory (supplementary material, Table S3).

In each of the five cases with metachronous lymphomas, the paired lesions shared an identical *IG* gene rearrangement (cases 2 and 4) and/or common somatic variants (2, 3, 21, and 23 variants in cases 1, 3, 5, and 6, respectively) confirming their clonal relationship (Table 1, Figure 2). In four cases, distinct variants were also seen between the paired lymphomas, indicating their divergent evolution from a clonally related lymphoma precursor (CLP) cell population. These included two cases (cases 1 and 2) with EMZL at both the initial and relapse presentations, one case (case 3) with primary EMZL but DLBCL relapse, and a further case (case 4) with DLBCL at both primary and relapse presentations. In each of these four cases, the initial thyroid lymphoma was successfully treated and achieved complete remission in cases 1, 3, and 4 or showed no clinical evidence of lymphoma after the open biopsy in case 2.

In the remaining case (case 5) with metachronous EMZL, both lesions harboured a *BCL6* rearrangement and a total of 21 common variants, with the later lesion showing an accumulation of further mutations over the initial lymphoma, in keeping with the partial regression of the original lymphoma following local radiation therapy (Figure 2).

In the case with composite EMZL and EBV‐positive DLBCL (case 6), both lymphomas harboured 23 common variants, including mutations in *CD274*, *TET2*, and *TNFRSF14*, and the EBV‐positive DLBCL showed an additional *MYC* translocation and a further 20 variants over the EMZL component (Table 1, Figure 2). Thus, the EBV‐positive DLBCL arose from the high‐grade transformation of an existing EMZL.

**Figure 3:**
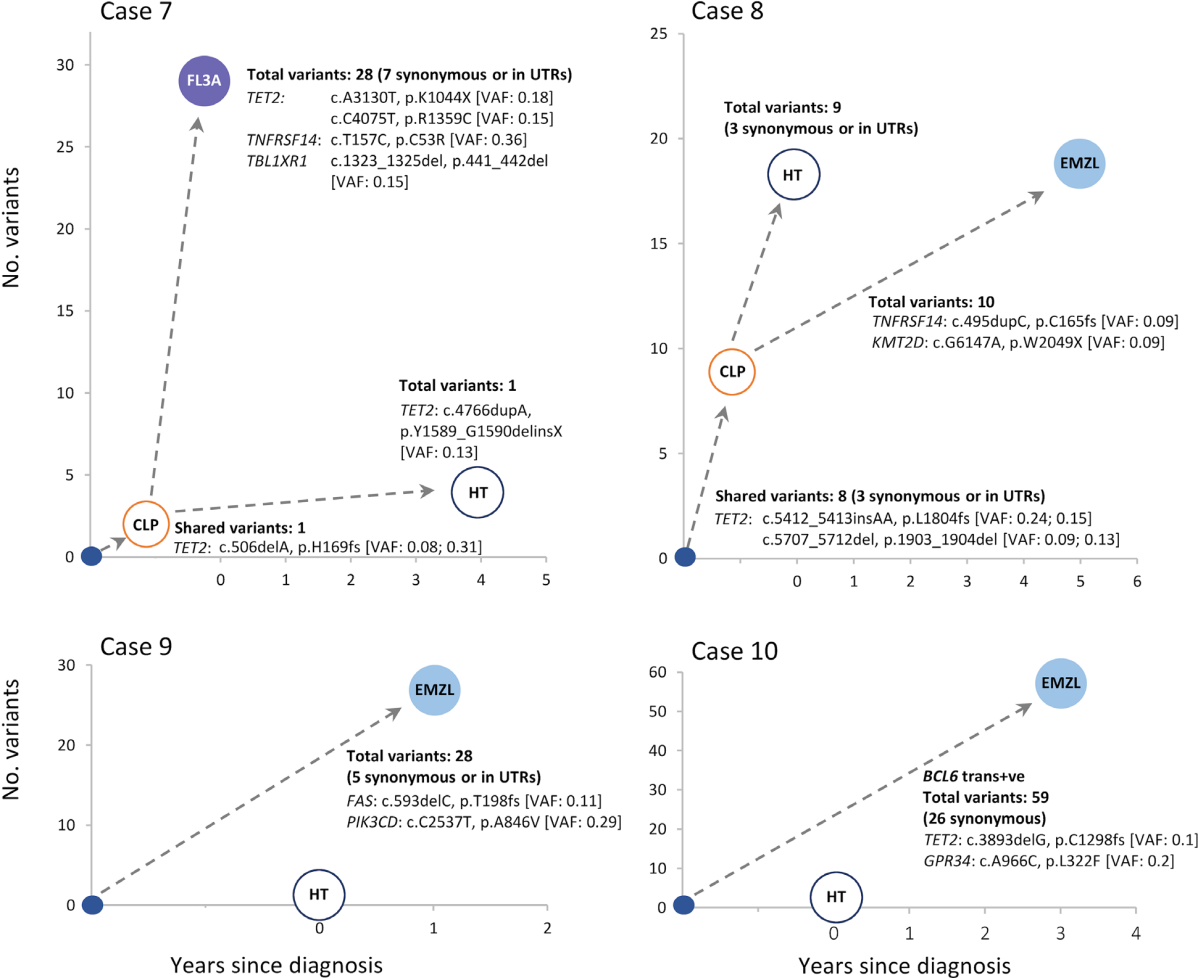
Variants in thyroid lymphomas and their paired HT lesion. The number of shared and distinct clonal mutations in paired lesions and their predicted evolutionary trajectory are indicated. The total number of all clonal variants (including pathogenic, benign, synonymous variants, and those in UTR regions) is given, but only representative pathogenic mutations are shown in detail. CLP, clonally related lymphoma precursor cells; EMZL, extranodal marginal zone lymphoma of mucosa‐associated lymphoid tissue; FL, follicular lymphoma; HT, Hashimoto's thyroiditis; trans+ve, translocation positive; VAF, variant allele frequency, for variants shared by paired lesions, and so thought to occur in CLP cells; the two VAF values in brackets correspond to the paired lesions in sequence.

The mutation profile in two of the four cases with paired lymphoma and HT biopsies indicated their divergent evolution from a CLP (Table 1, Figure 3). This included case 8, where an identical *IGK* gene rearrangement was demonstrated between the paired lymphoma and HT lesions. Regardless of the nature of paired lesions, the predicted CLP cells in all cases contained no or few (≤4) potentially pathogenic mutations, with the most frequently affected gene being *TET2*.

## Discussion

It is well established that a high proportion of transformed FL and relapsed FL may derive from the CLP clonally related to their original FL via divergent evolution. In this study, we demonstrated that a relapsed thyroid lymphoma may also develop via divergent evolution from a CLP cell, which was likely premalignant as indicated by the paucity of pathogenic mutations in cases 1, 3, and 4, though not in case 2.

There are important differences in the molecular events that drive clonal evolution of CLP between FL and thyroid lymphoma. In the former, the clonal evolution of CLP is primarily driven by the overexpression of BCL2 due to *IGH*::*BCL2*, which prevents the translocation‐positive cells from apoptosis while undergoing germinal centre reactions, whereas in the latter, the clonal evolution of CLP is most likely driven by dysregulated immune responses (autoimmunity). Nonetheless, in both scenarios, the prolonged clonal evolutionary process generates a ‘family’ of clonal cells, not only the subclone accounting for malignant transformation and lymphoma development, but also other subclones at various positions of the ‘family pedigree’. These subclones may continue the evolutionary process as long as the lymphomagenic microenvironment persists, which puts it at risk of acquiring further oncogenic events for malignant transformation.

The estimated relapse rate in patients with thyroid lymphoma is 6%–9% according to two large series of studies from Japan [13, 14], and this is far higher than the estimated incidence (0.37%) of primary thyroid lymphoma in patients with HT [15]. This further highlights the increased malignant potential of CLP in patients with thyroid lymphoma. Our observations also raise the question as to how relapsed lymphoma should be routinely investigated as its evolutionary trajectory can only be delineated by mutation profiling, not by conventional clonality analysis of the rearranged *IG* genes.

Among the potentially pathogenic changes predicted in CLP cells, *TET2* mutation is the most frequent, seen in three of the four cases with divergent evolution of metachronous lymphomas, as well as in two of those with prior or subsequent biopsy showing HT. These findings suggest that *TET* mutation is likely an early event. As shown in our previous study of 76 cases of thyroid EMZL [16], the VAF values of *TET2* mutation were similar to those of *TNFRSF14* and *CD274* mutations, although higher than those of *TNFAIP3* changes, potentially due to the homozygous nature of the *TNFAIP3* mutations in cases. In line with these previous observations, the current study also showed similar VAF values among *TET2* and other clonal mutations (data not shown). Together these findings suggest that *TET2* mutations might be a lymphoma clone‐specific event, not at the haematopoietic stem/progenitor cell level. Nonetheless, this requires further investigation of the corresponding *TET2* mutations in myeloid, non‐neoplastic B and T cells.

*TET2* encodes a dioxygenase, which catalyses the conversion of 5‐methylcytosine to 5‐carboxycytosine and promotes cytosine demethylation. Mice with B‐cell‐specific *Tet2* knockout appeared to experience a minor effect in B‐cell development [17, 18], but together with *Tet3* deficiency it caused spontaneous hyperactivation of both B and T cells and autoimmunity [19]. It remains to be investigated how *TET2* inactivation by mutation may reshape peripheral tolerance and impact the acquisition and selection of cooperative oncogenic changes in the genesis of thyroid lymphoma [17, 20].

## Author contributions statement

M‐MT, NW, ZC, FW, JM, EM, SG and M‐QD designed the experiments and collected and analysed the data. NW, KS, KI, ADA and AW contributed cases and undertook pathological assessment. M‐QD and M‐MT wrote and prepared the manuscript. M‐QD and NW designed and coordinated the study. All authors commented on the manuscript and approved its submission for publication.

## Supporting information


**Figure S1.** A sequencing coverage in each sample after deduplication and quality control filtering
**Figure S2.** Examples of somatic mutations identified by targeted next‐generation sequencing viewed on Integrative Genomics Viewer (IGV)

**Table S1.** Antibodies used for immunohistochemistry
**Table S2.** Genes investigated by TWIST capture and NGS
**Table S3.** Variants detected by targeted sequencing of 275 associated genes

## Data Availability

The data that support the findings of this study are available in the supplementary material of this article.
