# Supplementary material for: Primary thyroid B‐cell lymphoma: molecular insights into its clonal evolution and relapse
Source: J Pathol. 2024 Dec 26;265(2):123–31. doi: 10.1002/path.6380 (PMC11717488; doi:10.1002/path.6380)
Supplement: Supplementary file 1 — Figure S1. A sequencing coverage in each sample after deduplication and quality control filtering Figure S2. Examples of somatic mutations identified by targeted next‐generation sequencing viewed on Integrative Genomics Viewer (IGV) [file PATH-265-123-s002.docx]

**Primary thyroid B-cell lymphoma: molecular insights into its clonal evolution and relapse**

M-M Tzioni *et al. J Pathol* <https://doi.org/10.1002/path.6380>

**Supplementary Figures S1 and S2**

**Supplementary Tables S1–S3 are provided separately in Excel format**


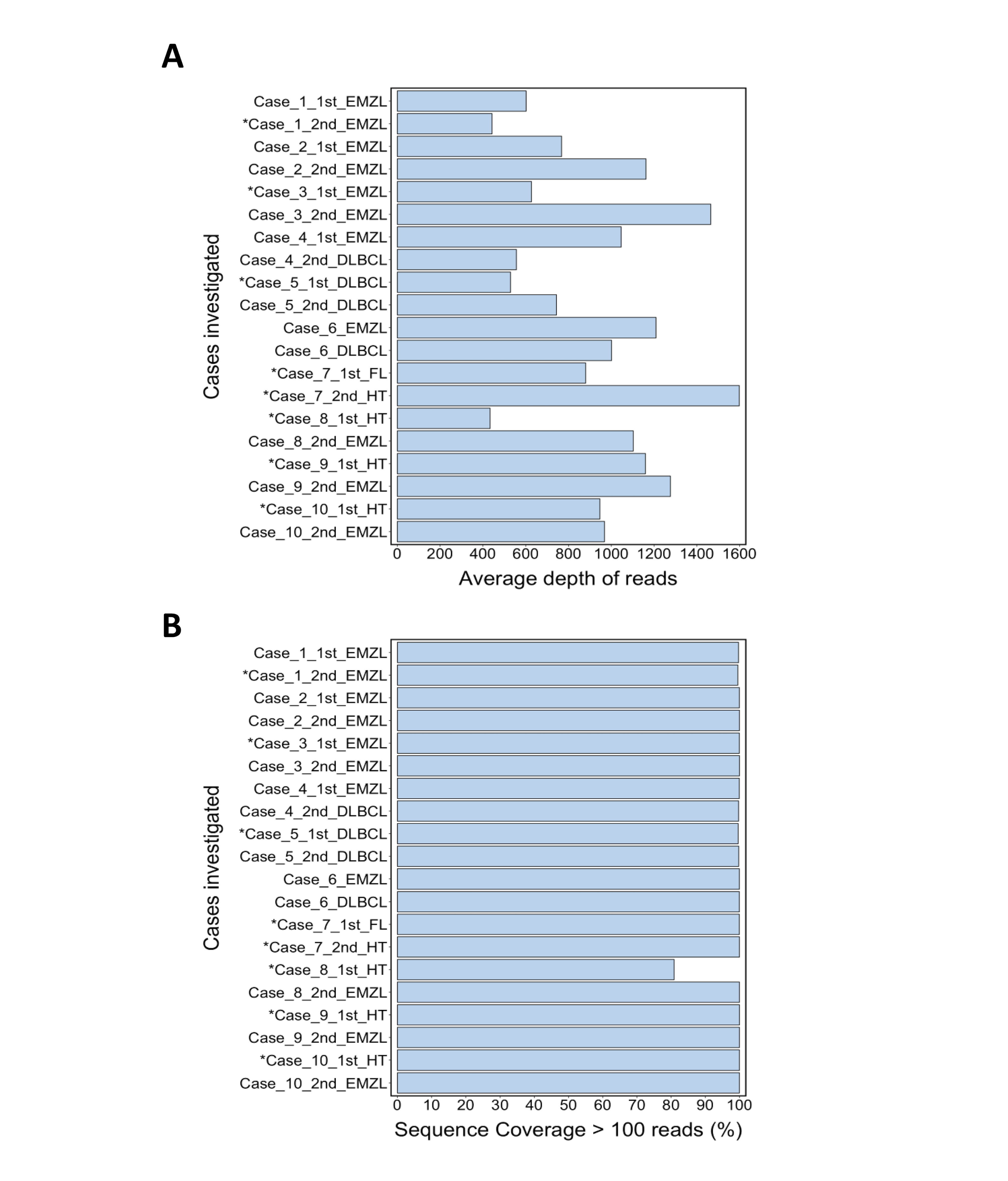


**Figure S1.** Sequencing coverage in each sample after deduplication and quality control filtering. Average depth of reads (A) and percentage of coverage >100 reads (B) for each sample investigated. *Specimens with suboptimal DNA quality were investigated in duplicate.


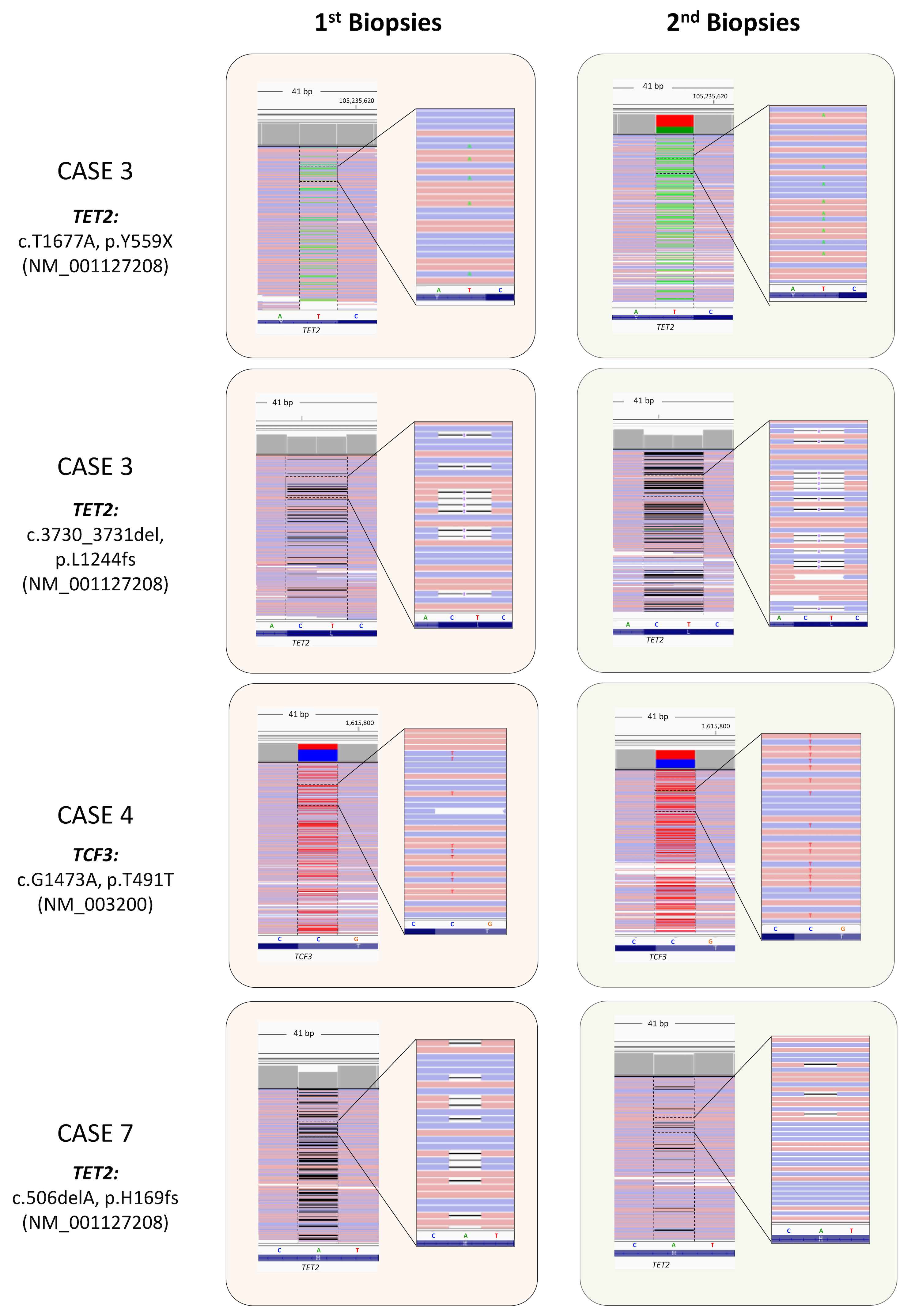


**Figure S2.** Examples of somatic mutations identified by targeted next-generation sequencing viewed on Integrative Genomics Viewer
